# Current Update on Role of Hesperidin in Inflammatory Lung Diseases: Chemistry, Pharmacology, and Drug Delivery Approaches

**DOI:** 10.3390/life13040937

**Published:** 2023-04-03

**Authors:** Salman Hosawi

**Affiliations:** 1 Department of Biochemistry, Faculty of Science, King Abdulaziz University, Jeddah 21589, Saudi Arabia; shosawi@kau.edu.sa; 2 Centre for Artificial Intelligence in Precision Medicines, King Abdulaziz University, Jeddah 21589, Saudi Arabia

**Keywords:** hesperidin, inflammation, respiratory lung diseases, flavonoids

## Abstract

Inflammation is a common feature of many respiratory diseases, such as pneumonia, asthma, pulmonary fibrosis, chronic obstructive pulmonary disease (COPD), lung cancer, acute lung injury, and COVID-19. Flavonoids have demonstrated their anti-inflammatory and antioxidant effects by influencing inflammation at different stages and majorly impacting several respiratory diseases’ onset and development. According to current studies, hesperidin, one of the most abundant polyphenols, can inhibit transcription factors or regulatory enzymes essential for controlling inflammation-linked mediators, including nuclear factor-kappa B (NF-κB), Inducible nitric oxide synthase (iNOS), and cyclooxygenase-2 (COX-2). It also improved cellular antioxidant defences by activating the ERK/Nrf2 signalling pathway. Therefore, this review provides the latest studies on the effect of hesperidin in different respiratory diseases, its pharmacokinetic profile, and innovative drug delivery methods.

## 1. Introduction 

Airway inflammation has a causative role in the pathophysiology of several significant respiratory diseases, including lung fibrosis, asthma, chronic obstructive pulmonary disease (COPD), acute respiratory distress syndrome (ARDS), pulmonary fibrosis, emphysema, and other respiratory diseases. Inflammation increases due to the production of pro-inflammatory cytokines, which cause mononuclear cells and polymorphonuclear eosinophils neutrophils to migrate into the lung tissue [1,2]. Over the last 10 years, many natural substances or phytochemicals with anti-inflammatory activities have been discovered as potential treatment possibilities. Additionally, drug development efforts focused on these natural compounds’ high efficacy and few adverse effects.

Hesperidin is a member of polyphenolic phytochemicals called flavonoids known for their wide range of pharmacological activities, such as anti-oxidative, anti-inflammatory, and anti-neoplastic properties [3,4]. Hesperidin is beneficial in treating and managing respiratory problems, as demonstrated by several clinical investigations [5]. In addition, hesperidin has been found to have a preventative impact against respiratory infections [6,7]. The primary purpose of this study is to provide readers with a thorough grasp of how hesperidin benefits conditions related to the lungs or the respiratory system. The central focus of this review is the significance of hesperidin in managing all lung disorders and the healing process.

## 2. Search Strategy

We searched PubMed/Medline, Science Direct, Scopus, ProQuest, and Google Scholar as well as Google databases using the following keywords: “hesperidin”, “drug delivery”, “inflammation”, “lung fibrosis”, “asthma”, “COPD”, “acute respiratory distress syndrome (ARDS)”, “pulmonary fibrosis”, “emphysema”, “Pharmacokinetics”, “toxicity”, “bleomycin”, and “oxidative stress.

Relevant English-language papers published up through December 2022 were eligible for inclusion in this analysis. This review comprised every paper that evaluated the impact of hesperidin on inflammatory lung disorders. This assessment excludes articles with minimal information, including letters and comments. In addition, research investigating the impact of naringenin on disorders other than those related to lung inflammation were excluded.

Two reviewers separately retrieved literature by title/abstract to choose eligible articles; studies that did not satisfy the eligibility criteria were eliminated. The entire texts of the shortlisted articles were next scrutinised for eligibility and data extraction. In situations of disagreement, debated articles were considered by writers, who then reached a conclusion.

## 3. Sources of Hesperidin

Hesperidin is present in citrus fruits, which include lemons, oranges, and several other vegetables and fruits. French scientist Lebreton initially isolated it, using the ellipticity (the spongy inside of the peel) of oranges in 1828 [8]. Citrus maxima, sweet oranges, and unshiu rinds have all been widely used to isolate them [9]. It has also been documented in other citrus species, including *Citrus reticulata Blanco* and *Citrus aurantium* L. *Var. Dulcis* (mandarin or tangerine) [10]. In addition, citrus species have higher concentrations of hesperidin in their albedo, membranes, and pith than in their seeds and juice vesicles. Furthermore, it has been claimed that the hesperidin content in green fruits changes as they mature. Furthermore, there is a significant connection between hesperidin levels and seed germination, demonstrating that light exposure increases the formation of this molecule (Table 1).

## 4. Chemistry

**Figure 1:**
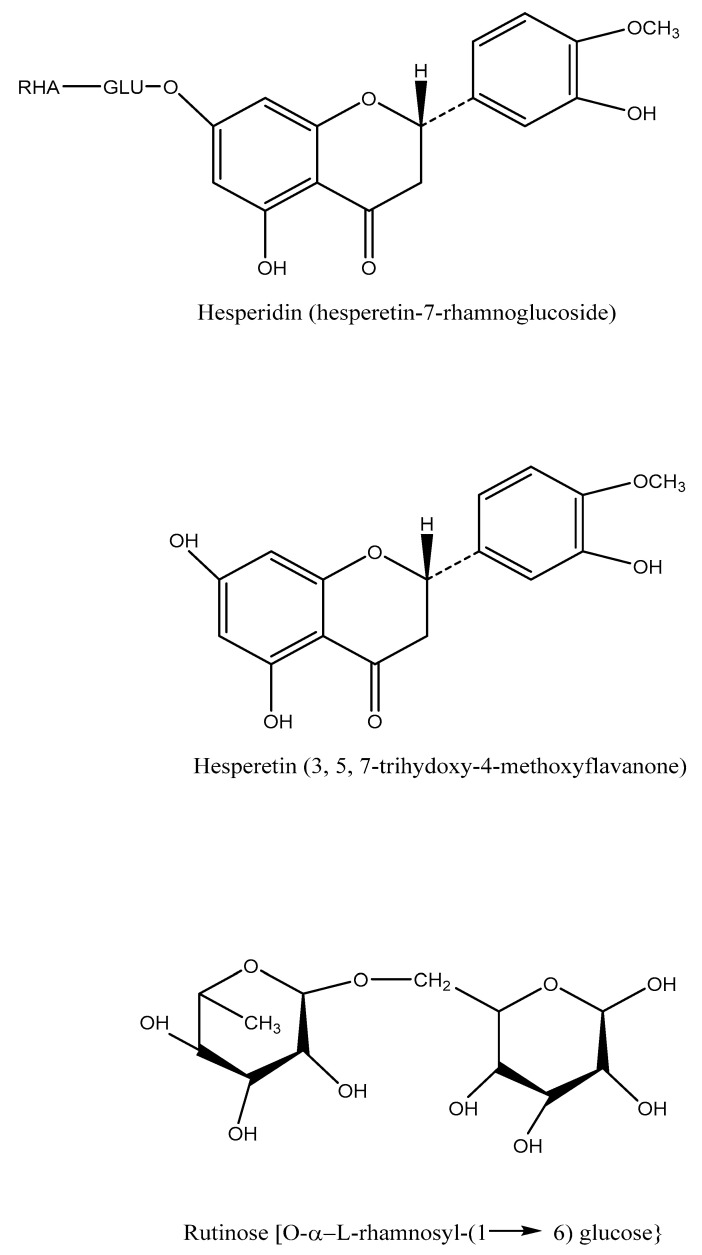
Structure of hesperetin and its derivatives.

An aglycone called hesperetin or methyl eriodictyol with an associated disaccharide called rutinose makes up the flavanone glycoside known as hesperidin. Therefore, hesperetin’s -7-rutinoside is called hesperidin. One molecule of glucose and one molecule of rhamnose comprise the disaccharide unit (C12H22O10), which may take on either of its two isomeric forms, neohesperidose or rutinose. Chemically, rhamnosyl-(1→6) glucose or 6-O-(6-deoxy-L-mannopyranosyl)-D-glucose make up rutinose. Chemically speaking, neohesperidose is O-L-rhamnosyl-(1→2) glucose, with the two sugar units’ arrangement being the sole difference. By partially hydrolyzing hesperetin with diluted acid to produce L-rhamnose and hesperetin-7-Dglucoside, by which the enzyme could break down -Dglucosidase, the location of the sweeteners in hesperidin was established. As a result, in hesperidin, rhamnose is connected to glucose and glucose is coupled to hesperetin. Chemically speaking, hesperetin (C_16_H_14_O_6_) is a 3,5,7-trihydroxy-4-methoxy flavanone. Hesperidin, following alkaline hydrolysis, gives phloroglucinol and hesperetin acid. Hesperidin is thus 3,5,7-trihydroxy-4-methoxy flavanone-7-(6-L-rhamnopyranosyl-D-glucopyranoside or -7-rutinoside [7] (Figure 1).

## 5. Physical Properties

Long, hair-like needles of pure hesperidin are tan or light yellow. It softens at 250 °C and has a melting point between 258 and 262 °C. Its chemical composition is C_18_H_34_O_15_, and its molecular weight is 610.57 daltons. Hesperidin is readily soluble in pyridine, providing a clear yellow solution, and in diluted alkali. It is virtually insoluble in acetone and marginally soluble in hot glacial acetic acid. Upon L-rhamnose, hesperetin, aglycone, and D-glucose are all produced in equal amounts by the hydrolysis of hesperidin. By hydrolyzing (±)- and (−)-hesperetin with either diluted sulfuric acid in ethylene glycol or sulfuric acid, an optically active mixture of (±)- and (−)-hesperetin is created, which may be divided by fractional recrystallization. Both share the trait of becoming derivatives. Hesperidin produces the well-controlled acid hydrolysis of the optically active laevorotatory aglycone (−)-hesperetin. This compound then produces L-malic acid upon ozonization, demonstrating that (−)-hesperetin possesses the 2S configuration. The disaccharide’s structure and the amount or lack of unpleasantness in the chemical are known to be correlated. Neohesperidosides are quite bitter, but rutinosides have no flavour. Due to its flavonoid rutinoside nature, hesperidin is not bitter in and of itself. While non-bitter rutinosides predominate in oranges and lemons, grapefruit mainly assembles bitter neohesperidosides [17,18].

## 6. Pharmacokinetics

Healthy white men (human volunteers) aged 25 were given 500 mg of the medication in water and equal volumes of orange juice and grapefruit to test the oral bioavailability of hesperidin from citrus products. It was taken from the digestive tract after the oral route in either form, but continuous urinary collection revealed low bioavailability. Hesperetin, an aglycone, was found in both plasma and urine. Absorbed before urinary excretion, citrus flavanones were assumed to go through glucuronidation [19]. To investigate membrane absorption of hesperidin derivatives, a cultivated layer of Caco-2 cells was used to represent the small bowel epithelium. Hesperidin does not penetrate the Caco-2 single layer, likely because of its lower solubility, but its glycosides do, and this penetration is dose-dependent. The paracellular route had been assumed to be the mechanism for this penetration [20]. The diet has been discovered to affect the rabbits’ ability to absorb hesperidin when given orally. When combined with a synthetic ration, it was absorbed, but not when combined with a standard pelleted feed. After oral administration to rats, flavonoids, including hesperetin and hesperidin, had their metabolic M-hydroxyphenyl propionic acid studied and smaller levels of m-coumaric acid in the aglycones were the common metabolic products found in urine. The aglycones were both free and glucuronic acid conjugated. This suggested dehydroxylation, demethoxylation, or demethylation coupled with dehydroxylation occurred after intestinal absorption, producing m—acid hydroxyphenyl propionic. According to research, hesperetin was more quickly absorbed than hesperidin in mice, rabbits, and humans [7]. Human volunteers consumed hesperidin, and a significant change in metabolism was seen. The main urinary metabolite, 3-hydroxy-4-methoxyphenylhydracrylic acid, showed that the breaks of the pyran circle of hesperetin produce hydracrylic acid. Additionally, a minor quantity of hesperetin’s glucuronide was found. The bacteria that produce endo-beta-glucosidase, beta-glucosidase, and alpha-mannosidase were shown to convert hesperidin to its hesperetin, aglycone, in the gut. Moreover, it was discovered that the metabolite produced in the human gut had stronger antiplatelet action and cytotoxicity than the parent molecule. The research was recently conducted on the plasma dynamics and urine outflow of hesperetin and naringenin from grapefruit and orange juice. Juices were administered to healthy participants, and plasma and urinary tests were taken and evaluated with HPLC. Hesperetin was shown to be available from the sample juices. However, there were significant inter-individual differences. Since the urine levels varied so much, it was determined that they could not be used as food consumption indicators [21]. Animal tests demonstrated that Daflon-500 mg (a mixture of fractionated flavonoids) almost completely disappeared 96 h after treatment, with no adverse buildup in any specific organ. Rat resorption and excretion research showed that a semi-synthetic derivative of natural hesperidin, a hydrosoluble, 14C-hesperidin methyl chalcone, absorbed 1–2 h after an oral delivery system at a dosage of 10 mg/kg body weight. The intravenous drug injection at the same dosage confirmed the entero-hepatic cycle predicted by the blood kinetic patterns. The blood profiles showed that the medication was bioavailable in a favorable way. Following oral consumption, fecal excretion was higher than urinary excretion, but the two were equivalent after intravenous treatment. Furthermore, excretion mostly happens during the first 24 h after treatment, regardless of the route [22].

## 7. Toxicity of Hesperidin

Hesperidin is a nontoxic flavonoid, and there are not many studies regarding its negative effects, even in pregnant women. In dosages between 0.3% and 5%, it is also classified as non-chemical. According to clinical studies, phosphorylated hesperidin (100–125 mg/kg/day) is utilized as an antifertility agent, and its antifertility effect is unaffected by trauma, infections, or systemic illnesses. Another clinical trial found that 10% of patients receiving Daflon 500 mg (hesperidin + diosmin) two times daily for six weeks to one year had mild side effects compared to those of the placebo group. Hesperidin may, however, interact with widely used medications such as daunomycin and vincristine, increasing vincristine’s absorption (10–50 M) across the BBB. Therefore, this research recommended avoiding hesperidin-containing meals while consuming vinblastin (especially P-glycoprotein substrates) [7]. Phosphorylated hesperidin (4%), readily digested and generating no adverse responses in animal experiments, is safe and non-toxic. Additionally, the oral bioavailability of methyl hesperidin (5%) has not been shown to have any cancerous or mutagenic effects on a rat. Moreover, consumption of hesperidin (0.3–5%) showed no side effects on food consumption, body weight, or food efficiency. Another research found that rats receiving methyl hesperidin (0.3–5%) for 13 weeks did not die, lose weight, or exhibit anomalies in their normal activities [23].

## 8. Role of Hesperidin in Inflammatory Lung Diseases

### 8.1. Asthma

Asthma, a primary noncommunicable disease (NCD) that affects both children and adults, is the most common chronic illness in children, and may include coughing, wheezing, and shortness of breath caused by inflammation, tightness in the chest, and constriction of the small airways in the lungs [24,25]. Citrus-farming yields hesperidin, a flavanone derivative of the flavonoid hesperetin and the sugars rutinose in large quantities and at a low cost. Using an allergic asthma model of the mouse, Dajun Wei et al. sought to ascertain if hesperidin inhibits ovalbumin-induced airway inflammation. OVA (ovalbumin) challenged and sensitized mice, resulting in persistent airway inflammation and remodeling. Compared to the mice exposed to OVA, hesperidin dramatically reduced the quantity of infiltration inflammatory responses and bronchoalveolar lavage fluid with Th2 cytokines. Hesperidin also decreased serum OVA-specific IgE levels. The OVA-induced airway hyperresponsiveness (AHR) to breathed methacholine was significantly reduced by hesperidin. According to lung histological examinations utilizing hematoxylin, eosin, and alcian blue-periodic acid-Schiff staining, hesperidin decreased hypersecretion, and inflammatory cell infiltration combined with the mice group was exposed to OVA. Their results explain the immune function of hesperidin in case it protects a mouse asthma model [26]. In another study, Hesperidin-3′-O-Methylether inhibits phosphodiesterase more effectively than hesperidin airway hyperresponsiveness brought on by ovalbumin: inhibition and suppression hesperidin and hesperidin-3′-O-effects methyl ethers on phosphodiesterase suppression and airway hyperresponsiveness (AHR) in an asthma model of the mouse were compared by Yang et al. According to their findings, hesperidin-3′-O-methyl ether significantly decreased the induced pause value and suppressed the total number of inflamed cells, including macrophages, eosinophils, neutrophils, and lymphocytes as well as complete and OVA-specific immunoglobulin (Ig)E levels in serum and BALF. It also increased the total quantity in mice’s serum sensitized to the substance and challenged with it. They found that hesperidin-3′-O-methyl ether has a greater therapeutic ratio and is effective in inhibiting phosphodiesterase and suppressing AHR. As a result, the potential for hesperidin-3′-O-methyl ether to be used in treating allergic asthma and COPD may be greater [27]. Hesperidin, a purported Th2 cytokine blocker, benefits asthma in a mouse model of allergic asthma.

**Figure 2:**
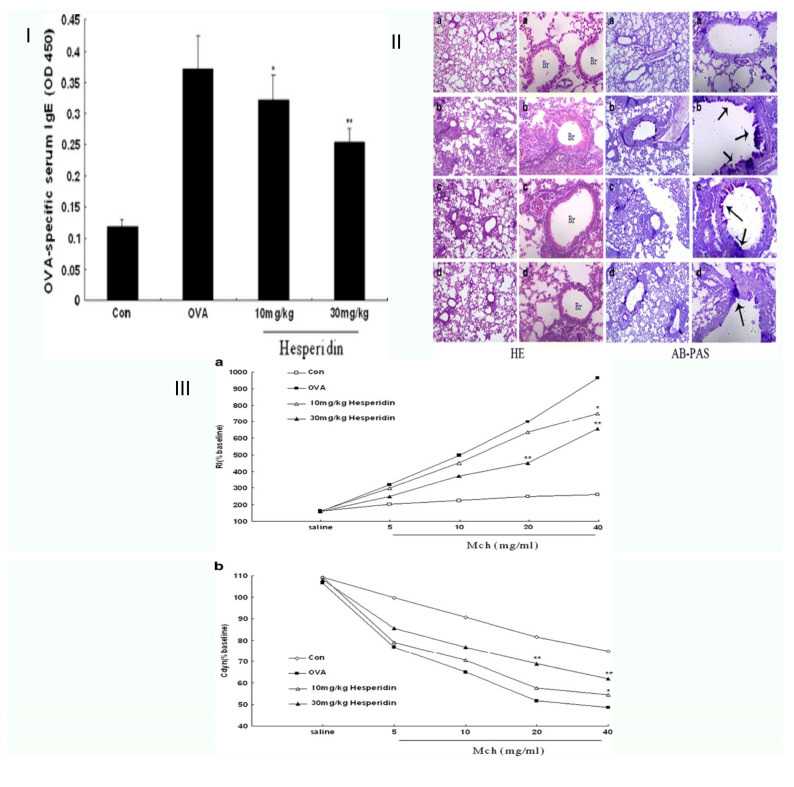
Schematic representation of hesperidin’s ability to reduce asthma symptoms in an allergic airway inflammation model. (**I**) Hesperidin’s (milligrams per kilogram) impact on serum OVA-specific IgE. (**II**) Representative hematoxylin–eosin and alcian blue-periodic acid-Schiff stained sections of lung from: (a) PBS-challenged mice; (b) OVA-challenged mice; (c) OVA-challenged mice treated with hesperidin (10 mg/kg); (d) OVA-challenged mice treated with hesperidin (30 mg/kg). The left panel is magnified 100×; the right panel is magnified 400×. Br bronchi, V vessel. Arrows indicate areas of alcian blue+cells. (**III)** In response to methacholine, hesperidin therapy decreased RI and restored Cdyn in OVA-challenged mice. Airway hyperresponsiveness was assessed by percentage change from the baseline level of (a) lung resistance (RI, n = 6 mice per treatment group) and (b) dynamic compliance The values represent the mean ± SEM of three independent experiments. * *p* < 0.05, ** *p* < 0.01. vs. OVA [26].

Seung-Hyung Kim et al. examined the anti-inflammatory and anti-asthmatic effects of hesperidin on the production of interleukin -17 (IL-17), eotaxin, and -OVA-specific IgE and Th2 cytokines. They looked at how hesperidin affected the generation of Th2 cytokines, OVA-specific IgE, pulmonary eosinophilic infiltration, different immune cell morphologies, and hyperresponsiveness of the airways in a mouse model of asthma. Results showed that hesperidin-treated groups reduced OVA-specific IgE, IL-5, and IL-17, reducing allergic airway inflammation levels, eosinophil infiltration, and AHR. Their findings revealed that hesperidin successfully cures asthma by decreasing the synthesis of eotaxin, OVA-specific IgE, eosinophil, and Th2 cytokines (IL-5) infiltration via the suppression of the GATA-3 transcription factor [24]. Later, Chang et al. investigated the anti-inflammatory effects of hesperidin and its mechanisms in an asthmatic murine model induced by ovalbumin, and looked into how hesperidin may affect the balance of Th1 and Th2 cells. Airway luminal constriction, the formation of airway hyper-responsiveness, an increase in eosinophils in bronchoscopy (BAL) fluid, and an increase in infiltration by lung tissue caused inflammation in cells surrounding airways and blood vessels were all symptoms of AHR. Hesperidin treatment before the last pulmonary OVA exposure significantly reduced all asthmatic symptoms. As a result, their research has shown that hesperidin is essential for improving the pathogenetic pathway of asthma in mice. Their results enhance existing views in comprehension of the immunopharmacological activities of hesperidin and provide unique insight into the immunopharmacological action of hesperidin as it relates to its effects in a mouse asthma model [25,26] (Figure 2).

### 8.2. COPD

A set of diseases known as a COPD includes chronic bronchitis and emphysema [21]. Hesperidin’s ability to reduce oxidative stress and inflammatory reaction in COPD rats may be connected to the PGC-1/NF-B signalling axis/ SIRT1. Shuyun Wang et al. studied the function of hesperidin in a COPD mice model to provide a foundation for using hesperidin. Cigarette smoke extract (CSE) was exposed to the mice to create COPD models. Hesperidin was then administered to the animals. The results demonstrated that inflammatory cell infiltrating and CSE brought on cell death in the mouse lung tissues, but hesperidin efficiently reversed these pathological alterations. Hesperidin may successfully enhance superoxide dismutase (SOD) and catalase (CAT) levels in bronchoalveolar lavage fluid (BLAF) while decreasing IL-6, malondialdehyde (MDA), and IL-8 in BLAF and the lung MPO content in COPD mice. Hesperidin was also shown to consistently increase PGC-1 and SIRT1 expression levels while decreasing the phosphorylation of p65 in COPD mouse models. Hesperidin used in large doses generally had a better impact on COPD mice. It was finally observed that hesperidin reduced inflammation and oxidative stress in CES-induced COPD mice and was linked to the SIRT1/PGC-1/NF-B signaling axis, suggesting a potentially novel approach to the treatment of COPD [27].

### 8.3. Pulmonary Fibrosis

The injury and scarring of lung tissue lead to the development of the lung ailment pulmonary fibrosis [28]. Hesperidin improves experiments on bleomycin-induced lung fibrosis through suppression of Smad3/MPK/ TGF-beta1 and NF-kB pathways, according to research by Zheng Zhou et al. When bleomycin (BLM) was administered intraperitoneally to Sprague–Dawley (SD) rats, pulmonary fibrosis was induced. Hesperidin was administered to rats, and different lung and BALF parameters were measured. Hesperidin dramatically reduced elevated myeloperoxidase levels, hydroxyproline, and oxide-nitrosative stress in the lung and BALF. It also reduced the expressions of lung Nrf2 and HO-1 that BLM upregulated, and the expressions of IL-1, TNF-α, collagen-1, TGF-β, IL-6, and Smad-3 were upregulated. According to a Western blot examination, hesperidin improved lung AMPK, NF-kB, IB, and PP2C-protein expression. Hesperidin also lessens BLM-induced pulmonary fibrosis by inhibiting the IB/NF-kB,/ TGF-1/MPK, Smad3, and oxide-inflammatory marker (HO-1 and Nrf2) pro-inflammatory marker (IL-1, TNF-α, and IL-6) pathways. This improves the variation of oxide-inflammatory markers (HO-1 and Nrf2) and pro-inflammatory indicators (TNF-α) [29].

In addition, Jiasen Guo et al. discovered that the natural substance, neohesperidin, suppresses TGF-1/Smad3 signaling and reduces lung fibrosis brought on by bleomycin in rats. They discovered that neo hesperidin reduced TGF-1-induced extracellular matrix formation, myofibroblast differentiation, and fibroblast migration, inhibiting TGF-1’s damage to alveolar epithelial cells. Additionally, they acquired in vivo proof that neo hesperidin therapy prevented bleomycin-induced lung damage, even in rats with preexisting pulmonary fibrosis. According to their research, neohesperidin may treat progressive pulmonary fibrosis since it may target a key signaling pathway and profibrogenic responses [30]. Histopathological research of hesperidin as a radioprotector towards radiation-induced lung damage was examined by Gholam Hassan Haddadi et al. Three groups of fifty rats were created. G1: No HES or radiation exposure (sham). G2: Thorax received beta-irradiation. HES was administered to G3 along with orradiation. Results from histopathology after 24 h revealed radiation-induced inflammation and the presence of more inflammatory cells than in G1. Compared to G2, the administration of hesperidin greatly reduced such an impact. When comparing G2 to G1, histopathological analysis revealed a substantial rise in alveolar thickness, mast cells, inflammatory cells, vascular thickness, pulmonary oedema, inflammation, and fibrosis. The research has shown that hesperidin is a strong radioprotector against radiation-induced lung injury in tissue of rats. Compared to G2, hesperidin greatly reduced the mast cells, inflammatory response, and fibrosis. Hesperidin administration reduced radiation fibrosis and radiation pneumonitis in the lung tissue [31].

Hesperidin’s effects on idiopathic pulmonary fibrosis were assessed using micro-computed tomography, histopathology, and a bleomycin-rat model. Due to its biochemical, anti-inflammatory, and antioxidant capabilities, Cemile Ayse Görmeli et al. examined the therapeutic potential of hesperidin against pulmonary fibrosis using histological, biochemical, and micro-CT investigations. Based on their research, they proposed that hesperidin’s biochemical, anti-inflammatory, and antioxidant characteristics might prevent BLC-induced lung fibrosis. The lungs of BLC-treated rats underwent obvious histological alterations. Infiltration of macrophages and lymphocytes, along with fibroblast growth, were shown to thicken the interalveolar septa in these rats. Histopathological alterations have been less extensive in the BLC+ hesperidin group than in the BLC group. Compared to the BLC group, the hesperidin therapy resulted in lower lipid peroxidation and higher antioxidant status. Additionally, data from histopathology and biochemistry were significantly positively correlated with micro-CT findings. It was also shown to effectively reduce the severity of BLC-induced lung damage, which was employed as a model for IPF [32].

Hesperidin was later studied to see whether it may treat pulmonary fibrosis by preventing lung fibroblast senescence by Di Han et al. They showed that hesperidin could help mice with lung fibrosis brought on by BLC. Hesperidin therapy in vitro and in vivo dramatically downregulated the amount of senescence-associated galactosidase-positive cells by downregulating the expression of senescence signature proteins p53, p21, and p16 as well as the myofibroblast marker-SMA. Hesperidin, meanwhile, may block the IL6/STAT3 signaling pathway. Further research revealed that when the IL-6/STAT3 signaling pathway was blocked in vitro with LMT-28 pre-treatment, the inhibitory impact of hesperidin on fibroblast senescence appeared to fail [33].

**Figure 3:**
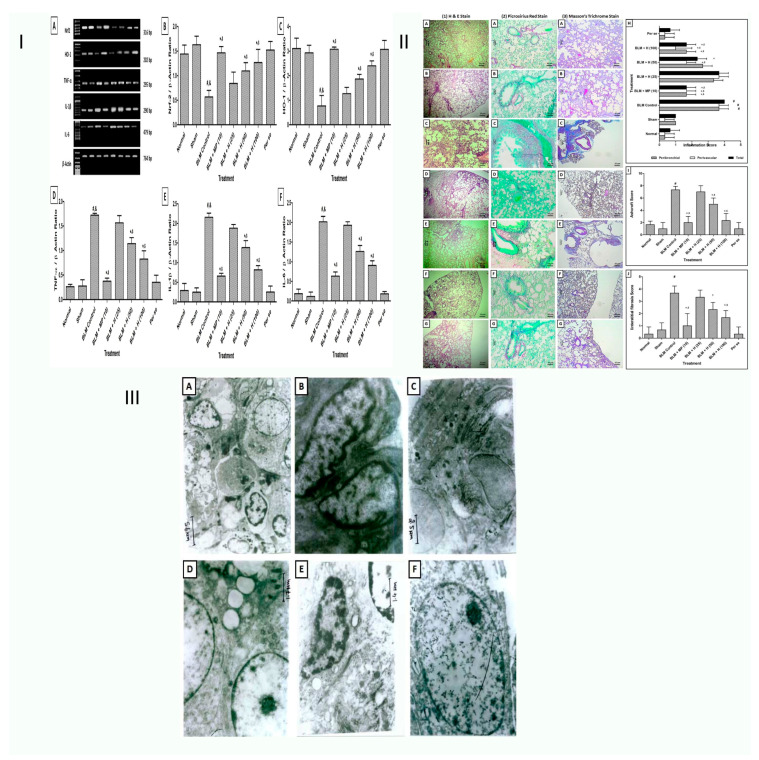
Schematic representation of hesperidin bleomycin-induced experimental pulmonary fibrosis. (**I**) Hesperidin’s impact on modifications in rat lung Nrf2, HO-1, TNF-β, IL-1, and IL-6 mRNA expression brought on by BLM. (A) on day 14, quantitative representation of mRNA expression of Nrf2 (B), HO-1 (C), TNF-α (D), IL-1β (E) and IL-6 (F). *p* < 0.05 when compared with: sham group (&), normal group (#), each other (MP or hesperidin) group ($), and BLM control group (*). (**II**) Effect of hesperidin on BLM-induced modifications in the rats’ lungs’ and airways’ histology. This is magnified 400×. Photomicrograph of sections of lungs of normal (A), sham control (B), BLM control (C), MP (10 mg/kg) treated (D), hesperidin (50 mg/kg) treated (E), hesperidin (100 mg/kg) treated (F) and Perse treated (G) rats. Lung H&E staining (1A–1G) on day 14, Lung Picro-Sirius red staining (2A–2G) on day 28 and Lung Masson’s trichrome staining (3A–3G) on day 28. Effect of hesperidin on alterations induced by BLM in lung airway inflammation score (H), Ashcroft Score (I) and interstitial fibrosis score (J). *p* < 0.05 when compared with: sham group (&), normal group (#), each other (MP or hesperidin) group ($), and BLM control group (*). (n = 3) (**III**) BLM altered rats’ lung ultrastructure, which was inhibited by hesperidin. Photomicrographs of lung from representative animals, normal (at 3474×) (A), sham control (at 11,580×) (B), BLM control (at 7720×) (C), methylprednisolone (10 mg/kg) treated (at (11,580×) (D), hesperidin (100 mg/kg) treated (at 13,510×) (E), and per Se treated rats (at 11,500×) (F) [34].

Rezaeyan et al. sought to determine the male rat lung tissue damage caused by irradiation and the radioprotective efficiency of hesperidin. As a radiosensitive organ, the lung, radiation doses must consider this. Their findings indicated that oral treatment of hesperidin protected against -irradiation-induced oxidative stress and pulmonary damage in rats. This protection was most likely provided by hesperidin’s ability to stabilize membranes and scavenge free radicals, which may have a beneficial impact on respiratory diseases [34] (Figure 3).

### 8.4. Lung Cancer

When lung cells divide too quickly, tumors form and the condition is known as lung cancer [35]. The worldwide burden of lung cancer is high; up to 18% of cancer-related fatalities are attributable to lung tumors. In terms of prevalence, lung cancer ranks second, and experts worldwide have made enormous attempts to treat it. Tumors may damage one’s ability to breathe, as well as have the possibility of spreading to other parts of the body [36]. Natural products are a major source of anti-tumour medications. Citrus sinensis Osbeck, a Rutaceae plant lime, contains the flavanone known as hesperidin. It is thought to reduce the viability of cancer cells in culture. However, hesperidin’s impact on lung cancer and its possible mechanisms are unknown. It was discovered that pinx1 expression level has a significant role in controlling invasion, cell proliferation, lung cancer senescence, and migration, and is strongly connected to overall survival. More notably, hesperidin markedly enhanced pinx1 protein expression, and pinx1’s specific siRNA prevented hesperidin’s protective effects. Additionally, it was determined that hesperidin is effective in vivo. The results demonstrated that hesperidin is a promising therapy for halting lung cancer development [35].

The carotenoid cryptoxanthin and the flavonoid hesperidin have inhibitory activity on carcinogenesis in various organs. Takuji Tanaka and colleagues, by using juice from a satsuma mandarin (MJs), created citrus juices with high levels of β-cryptoxanthin and hesperidin. Hesperidin and cryptoxanthin are present in significant concentrations. They showed that cationized peroxidase (CHRP) and MJs inhibit medically induced mouse lung, rat colon, and rat tongue carcinogenesis. Rats’ liver, tongue and colon detoxifying enzyme activity were increased after CHRP gavage.

Additionally, the target tissues’ production of pro-inflammatory enzymes and cytokines might be reduced by CHRP and MJs. Their research shows that orally ingested CHRP and MJs block the growth of epithelial neoplasms in mouse colon, lung, and tongue cancers by acting on various biological processes. Further, they concluded that CHRP and MJs might be used as cancer chemopreventive agents against the growth of tongue, colon, and lung cancer due to their ability to control proliferation and the mRNA expression of many cytokines and inflammatory enzymes, as well as their ability to prevent chemically induced detoxification enzymes and carcinogenesis [36].

Citrus unshiu Marc juice (MJ) and MJ5, especially MJ5 and MJ2, completely inhibited colon cancerogenesis in rats brought on by chemicals, according to Hiroyuki Kohno et al.’s study on the β-cryptoxanthin and flavonoids-rich mandarin juice and inhibitory impact on the compound 4-(methylnitrosamino)-1-(3-pyridyl)-1-butanone. MJ’s therapy reduced the number of lung neoplasms with no statistically significant differences. MJs, particularly MJ5, immunohistochemically decreased lung cancers with a PCNA-positive index (proliferating cell nuclear antigen). Hyperplastic alveolar cell lesions were unaffected. According to their results, MJ5 can chemoprevention 4-(methyl-nitrosamine)-1-(3-pyridyl)-1-butanone (NNK)-induced mouse lung carcinogenesis [37].

Later, Sattu Kamaraj et al. investigated the anticancer and antioxidant hesperidin’s involvement in benzo (a) pyrene-induced lung cancer in mice. Hesperidin’s ability to prevent lung cancer caused by B(a)P exposure in Swiss albino mice was investigated in this research. B(a)P administration to mice caused an increase in lung-specific tumour marker and lipid peroxides (LPO), serum marker enzymes, gamma-glutamyl transpeptidase (GGT), 5′nucleotidase (5′ND), lactate dehydrogenase (LDH), carcinoembryonic antigen (CEA), and aryl hydrocarbon hydroxylase (AHH), along with a concurrent reduced in the levels of tissue antioxidants such as superoxide dismutase (SOD), Vitamins E and C, peroxidase (gpx), reduced glutathione (GSH), and glutathione catalase (CAT). Treatment with hesperidin dramatically reduced these changes, demonstrating a strong anticancer impact on lung cancer. Additionally, proliferating cell nuclear antigen (PCNA) immunofluorescent and histological investigation supported hesperidin’s antiproliferative action. The results support hesperidin’s chemopreventive ability against chemically generated lung disease in mice [38].

### 8.5. Acute Lung Injury or ARDS

Acute lung injury (ALI) and the more severe immediate respiratory distress syndrome are pulmonary manifestations of an acute systemic inflammation process, including hypoxemia, pulmonary infiltrates, and oedema (ARDS) [39]. It was shown by Xin-xin Liu et al. that high-mobility group box 1 (HMGB1) release might be decreased to lessen the acute lung damage caused by lipopolysaccharide in mice. The investigation aimed to determine how HMGB1 is related to HDN-induced immunoregulation of ALI. LPS was administered intravenously to male BALB/c mice, where it caused ALI. Reduced lung wet-to-dry weight ratio increase, total cells, neutrophils, myeloperoxidase (MPO) activity, macrophages, and lower histological lung damage are signs that HDN substantially protected rats against LPS-induced ALI. The production of chemokines and pro-inflammatory cytokines, such as monocyte chemoattractant protein-1, interleukin-6 (IL-6), and, as tumour necrosis factor (TNF), were significantly reduced by HDN pre-treatment in the meanwhile (MCP-1). Additionally, HDN pre-treatment significantly reduced macrophage infiltration and lowered both HMGB1 release and expression in vivo and in vitro. Moreover, exogenous HMGB1 used intranasally might cause lung damage, which was likewise mitigated by HDN treatment. According to the findings, HDN treatment again shields mice against ALI-induced LPS by reducing TNF and IL-6 production. Additionally, they discovered that HDN might prevent the synthesis of MCP-1 and the infiltration of macrophages, inhibiting the expression and release of HMGB1 [37].

Hesperidin (HES) modulates local immunological responses in the lung in reaction to in vivo acute lung inflammation caused by LPS, according to research by Chia-chouyeh et al regarding the immunomodulation of both in vivo and in vitro endotoxin-induced acute lung injury. Mice were given intratracheal lipopolysaccharide, and hesperidin administration reduced the production of IL-6, KC, MCP-1, IL-12, MIP-2, and IL-1; the LPS caused this. Additionally, it increased IL-4 and IL-10 production. Hesperidin dramatically reduced inos expression, nitric oxide generation and proteins, and total leukocyte counts. HES reduced the production of THP-1 cells and IL-8 on A549 cells, IL-1 THP-1 cells IL-1, and VCAM-1 on A549 cells, all of which have an impact on the function of cell adhesion. IB and MAPK pathways stimulate NF-B and AP-1, which then regulate the repression of those molecules. Because HES blocks those pathways, the production of IL-8 and VCAM-1, IL-6, and CAM-1 is reduced. According to the research, HES significantly modulated the immune system in an important clinical ARDS model. However, further research is needed to assess the potential therapeutic value of HES in the supplementary treatment of ARDS [40].

**Figure 4:**
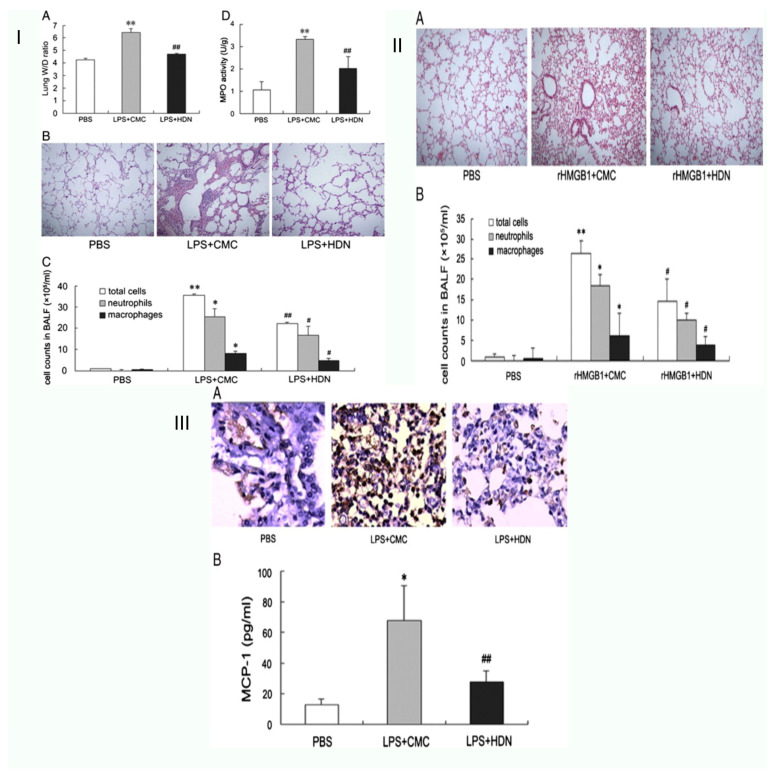
Schematic representation of hesperidin reduces the acute lung damage brought on by lipopolysaccharide in mice. (**I**) Pre-treatment with hesperidin reduces acute lung damage brought on by LPS. (A) The lung wet to dry weight (W/D) ratio determined 24 h after LPS challenge. (B) Hematoxylin and eosin staining of lung specimens 24 h after LPS administration (H&E staining, original magnification × 100). (C) The cell counts in BALF 24 h after LPS administration. (D) The MPO activity in lung tissues 24 h after LPS challenge. ** *p* < 0.01 versus the PBS group, * *p* < 0.05 versus the PBS group; ## *p* < 0.01 versus the LPS + CMC group, # *p* < 0.05 versus the LPS + CMC group. (**II**) Pre-treatment with hesperidin reduces HMGB1 release and expression brought on by LPS. (A) Hematoxylin and eosin staining of lung specimens collected 24 h after rHMGB1 exposure (H&E staining, original magnification × 100). (B) The cell counts in BALF collected 4 h and 24 h after rHMGB1 exposure. The values are presented as means ± SD (n = 6–8 in each group). ** *p* < 0.01 versus the PBS group, * *p* < 0.05 versus the PBS group; # *p* < 0.05 versus the rHMGB1 + CMC group (**III**) In LPS-induced ALI, hesperidin pre-treatment reduces macrophage infiltration. (A) Lung samples were collected 24 h after LPS administration for immunohistochemistry staining of macrophage (original magnification × 400). (B) BALF samples were collected at 4 h to determine the level of MCP-1. Data are expressed as means ± SD (n = 6–8 in each group). * *p* < 0.05 versus the PBS group; ## *p* < 0.01 versus the LPS + CMC group [42].

Ana Beatriz Fariasde Souza et al. postulated the results of hesperidin treatment in a possible mechanism of acute lung inflammation both in vitro and in vivo, where they assessed cell survival and production of reactive oxygen species in macrophages using various hesperidin doses. Hesperidin, they found, did not affect cell survival, but it did decrease intracellular ROS generation in cells treated with lipopolysaccharide (LPS). They also examined the effects of hesperidin in vivo in mice given the measles virus (MV). Animals given MV had greater macrophage, lymphocyte, and neutrophil counts in their bronchoalveolar lavage fluid, which suggested an inflammatory response. Although the antioxidant enzyme activity was reduced, MV caused oxidative damage and elevated myeloperoxidase activity in the lung tissue. The findings show that pre-treatment with hesperidin may protect infected mice’s lungs to respiratory support by regulating the inflammatory process and redox disequilibrium, and may work to prevent MV harm [41] (Figure 4).

### 8.6. Pneumonia

The air sacs in one or both lungs might become inflamed due to pneumonia. The air sacs may enlarge with liquid or pus (purulent material), which might cause fever, breathing problems, cough, and chills that produce pus [41,43]. Numerous species, such as fungi, viruses, and bacteria, may cause pneumonia. Hassan Haddadi et al. investigated the radioprotective effects of hesperidin on radiation-induced lung injury. Three groups of fifty rats were created to prevent radiation-induced lung damage in rats. G1: no hesperidin or radiation exposure (sham). G2: Thorax received beta-irradiation. Hesperidin was administered to G3 along with irradiation. A considerable rise in inflammatory cells, mast cells, vascular thickness, inflammation, alveolar thickness, pulmonary oedema, and fibrosis were seen after an eight-week histopathological assessment. Hesperidin administration reduced radiation fibrosis and radiation pneumonitis in the lung tissue. As a result, the research demonstrated that hesperidin is a powerful radioprotector in radiation-induced lung injury in tissue rats [31].

### 8.7. COVID-19

The viral disease known as coronavirus is caused by the SARS-CoV-2 virus (COVID-19) [42,44]. Canrongwu and others, by using computational approaches, analyzed the pharmaceutical targets for SARS-CoV-2 and found promising medications. Several naturally occurring substances and their derivatives with anti-inflammatory properties also demonstrated a binding affinity high too, 2-hydroxy-3,4-seco-friedelolactone-27-oic acid, with several derivatives of andrographolide, chrysin-7-O-glucuronide from isodecortinol, Cassine xylocarp, and cerevisterol from hesperidin, neohesperidin, kitchen side I, and deacetylcentapicrin from *Citrus aurantium*, *Viola diffusa*, and the *Swertia* plant species. The findings imply that these small-molecule substances may be suitable 3clpro inhibitors and useful in managing SARS-CoV-2 [45] (Table 2).

## 9. Drug Delivery in Respiratory Diseases

Ultimately, nanotechnology could be the most creative and successful strategy to increase bioavailability. When some substances are covered with a coating of nanoparticles, their permeability and the amount of that substance that may enter the bloodstream are boosted [50]. Healthy cells seem to be only marginally harmed by the nanoparticle covering. Nanotechnology research has also been conducted to improve hesperidin’s bioavailability. The antimicrobial zinc oxide nanostructures influenced by in silico and hesperidin direct comparison among antiviral phenolics for anti-SARS-CoV-2 purposes were studied by Gouda H. Attia et al. These natural medications and dietary supplements may be more affordable, accessible, safe, and have fewer adverse effects. This research compares ten phenolic antiviral drugs in silico against SARS-CoV-2 and identifies the potent metabolite of natural sources. Then, as a reducing agent, these metabolites were used to create zinc oxide nanoparticles (ZnO NPS). Every tested substance exhibited the anticipated anti-SARS-CoV-2 action. Hesperidin had a high docking score, so they isolated it from the peels of orange and used conventional spectroscopic research to establish its structure.

Moreover, IR, UV, XRD, and TEM were used to characterize the production of zinc oxide nanoparticles of hesperidin. Hepatitis A, an example of an RNA virus, was used to test the in vitro antibacterial activities of hesperidin and ZNO NPS. Hesperidin demonstrated an antiviral effect against HAV, although ZnO NPS did so more effectively. Therefore, more research against SARS-CoV-2 is necessary before using hesperidin and its linked ZnO nanoparticles as a viable therapeutic [51].

An effective biocompatible, phagocytosis, anti-cancer, anti-inflammatory-inducing model was examined by Ghassan M. Sulaiman et al. A medication delivery method based on hesperidin encapsulated on gold nanoparticles was explored. Using a chemical synthesis process, hesperidin packed on gold nanoparticles (Hsp-aunps) was created. The synthesis of Hsp-aunps was confirmed using various characterization methods, including Zeta potential measurement, XRD, FESEM, FTIR, EDX, TEM, and particle size analysis. The MTT and crystal violet tests evaluated the cytotoxic impact of Hsp-aunps breast cancer cells of the human line. Compared to a human breast epithelial cell line with normal proliferation, the findings showed that the treated cells’ growth was significantly inhibited (HBL-100). Acridine orange-propidium iodide dual stained test was also used for fluorescence microscopy apoptosis determination. The in vivo experiment aimed to assess the hazard of Hsp-aunps in rats. The indicators for kidney and liver function were evaluated. For the examined indicators, no statistically significant changes were discovered. After receiving therapy with Hsp-aunps, histological pictures of the lung, liver, spleen, and kidney revealed no obvious damages or abnormalities. Hsp-aunps improved macrophages’ functional efficacy against mice carrying ehrlich ascites tumour cells. It was also determined if bone marrow-derived cells treated with Hsp-aunps produced the pro-inflammatory cytokines. The findings showed that treatment with Hsp-aunps greatly reduced the release of IL-6, TNF and IL-1 [52].

Later, Hua Jin et al. conducted a study titled “Nasal Distribution of Hesperidin/Chitosan Nanoparticles Decreases Cytokine Storm Syndrome (CSS) in an Animal Model of Acute Lung Injury.” It showed that CSS, also known as cytokine storm, is linked to a high mortality rate in individuals with (ARDS) and lung injury (ALI). However, no proven therapies for ALI or ALI/ARDS caused by CSS exist. Therefore, there is still a pressing need to create potent medications and treatment approaches to combat CSS and ALI/ARDS. Due to its capacity to enhance medication delivery to the lungs, inhaled drug delivery techniques provide a potential option for managing respiratory disease.

Another potential approach in the battle against ALI/ARDS is increasing the nasal mucosa intake of weakly water-soluble medications with low mucosa solubility to a therapeutically useful level. Hesperidin-loaded chitosan nanoparticles (HPD/NPS) were created in this study for nasal administration of the anti-inflammatory HPD chemical to inflamed lungs. Compared to free HPD, HPD/NPS showed improved cellular absorption in the inflammatory milieu both in vitro and in vivo. Compared to free HPD, the HPD/NPS significantly lowered inflammatory cytokine levels and restricted vascular permeability in an animal model of inflammation lung disease, preventing lung damage. Their research shows that nasal HPD/NP administration lowers CSS in a chronic lung illness rat model. That anti-inflammatory nanoparticle-based therapeutic approaches may be employed to lower CSS and ALI in individuals with inflammatory lung damage [49].

## 10. Conclusions

In conclusion, hesperidin is a therapeutic, naturally occurring anti-inflammatory flavonoid that is abundantly present in various plants and vegetables. In review, we showed that hesperidin has anti-inflammatory, antiapoptotic, and protective roles in lung cancer, pulmonary fibrosis, COPD, pneumonia, and COVID-19. In addition, we reviewed hesperidin’s chemistry, pharmacokinetics, and preclinical pharmacological features, all of which might be useful in planning future clinical studies. Due to the significance of innovative drug delivery methods, we also suggested novel administration formulations and nanotechnology-based drug delivery systems, which may be given greater attention in hesperidin research in the future. Based on sufficient data, hesperidin may likely be seen as an alternate flavonoid medication for respiratory disorders. Studies using experimental animal models show that hesperidin significantly reduced organ damage and lung dysfunction.

Additionally, these results imply that it is possible to use the information from hesperidin-based animal research to develop a treatment approach for human lung disorders, namely pulmonary fibrosis and lung cancer. Furthermore, hesperidin may be used in experimental models of various respiratory conditions such as COPD, ALI, and asthma. Interestingly, these lung injuries have several characteristics, including tissue remodeling, oxidative stress, and inflammation, which are crucial therapeutic targets for hesperidin-mediated pulmonary protection. Furthermore, hesperidin’s nontoxicity and natural status as a substance with an outstanding safety record are further benefits. However, further research is necessary to evaluate hesperidin at different phases of illness development. In addition, its mechanism(s) leading to considerable protection still needs to be clarified to offer specific recommendations for assessing hesperidin in patients with respiratory disorders. Finally, as our understanding of the hesperidin processes grows, various therapeutic modalities may be used to improve hesperidin’s effectiveness in treating human respiratory illness. 

## Figures and Tables

**Table 1:** Natural sources and quantity of hesperidin.

Citrus Fruit Juices	Quantity	References
Grapefruit concentrate juice	1.55 mg/100 mL	[11]
Pure grapefruit juice	0.65 mg/100 mL	[11]
Juice from the concentrate of lemon	24.99 mg/100 mL	[12]
Lemon juice, pure	17.81 mg/100 mL	[12]
Pure juice, lime	13.41 mg/100 mL	[12]
Orange [Blond], concentrate juice	52.68 mg/100 mL	[13]
Pure orange [Blond] juice	25.85 mg/100 mL	[13]
Orange [Blond], concentrate juice	51.30 mg/100 mL	[13]
Orange [Blond] juice, undiluted	43.61 mg/100 mL	[13]
Tangerine, concentrate juice	36.11 mg/100 mL	[14]
**Herbs**	**Quantity**	**References**
Dried, peppermint	480.65 mg/100 g FW	[15]
Fresh welsh onion	0.02 mg/100 g FW	[16]

**Table 2:** Summarizes the role pharmacological role of Hesperidin in various respiratory lung diseases.

Respiratory Diseases	Model	Aim	Findings	References
Asthma	OVA-challenged mice	Allergic asthma model of the mouse. Hesperidin downregulated ovalbumin-induced inflammation of the airways.	Hesperidin suppressed inflammatory cell infiltration and mucus hypersecretion, decreasing OVA-specific IgE levels.	[26]
	OVA-induced lung eosinophilia and mucus hypersecretion in a mouse model of asthma	Hesperidin-3′-O-Methylether inhibits phosphodiesterase more effectively than hesperidin airway hyperresponsiveness brought on by ovalbumin: inhibition and suppression	It decreased the blood levels of OVA-specific immunoglobulin (Ig)E and the total number of macrophages, macrophages, neutrophils, lymphocytes, and eosinophils.	[27]
	Eotaxin, IL-17, and OVA-specific IgE in vivo asthma model mice	Hesperidin, a prospective Th2 cytokine antagonist, has anti-asthmatic effects in a mouse model of asthma.	HPN efficiently cures asthma by inhibiting the transcription factor GATA-3, which lowers the development of the eotaxin and Th2 cytokines (IL-5).	[46]
	Asthmatic mouse model induced by ovalbum	Hesperidin’s anti-inflammatory properties and its underlying mechanisms in a mouse model of asthma induced by ovalbumin	Decreased serum OVA-specific immunoglobulin (Ig)E levels, total inflammatory cell counts, macrophages, lymphocytes, neutrophils, and eosinophils, considerably reducing all asthmatic symptoms.	[26]
COPD	Cigarette smoke-exposed mice	Hesperidin’s ability to reduce oxidative stress and inflammatory reactions in mice having COPD may be connected to the SIRT1/PGC-1/NF-B signalling axis.	In mice with COPD caused by CES, hesperidin reduced the inflammatory and oxidative stress responses.	[47]
Pulmonary Fibrosis	Sprague–Dawley rats	By inhibiting the TGF-beta1/Smad3/AMPK, kappa alpha/NF-kappa B, and bleomycin-induced experimental lung fibrosis pathways, hesperidin reduces the severity of the condition.	Hesperidin reduces the effects of BLM-induced IPF by inhibiting the IB/NF-B, TGF-1/AMPK/Smad3 and oxide-inflammatory marker (HO-1and Nrf2) pro-inflammatory marker (TNF-, IL-6 and IL-1,) pathways.	[29]
	Mice	Neohesperidin reduces bleomycin-induced lung fibrosis in rats and suppresses TGF-1/Smad3 signalling.	Lowered the formation of extracellular matrix, fibroblast migration, and myofibroblast differentiation caused by TGF-1.	[30]
	Tissue damage in the lung of male rats by induced radiation	Analyzing hesperidin’s radioprotective effect on acute radiation damage in rat lung tissue	In rats, oral treatment of HES was observed to protect against irradiation-induced pulmonary damage. This protective action against inflammatory diseases is likely a result of HES’s capacity to scavenge free radicals and stabilise membranes.	[34]
	Radiation-induced lung injury of male rats	Hesperidin as a radioprotector regarding radiation-induced lung damage in rat	HES dramatically reduced lung tissue fibrosis, inflammation, and mast cell proliferation. It also reduced radiation fibrosis and radiation pneumonitis.	[31]
	Bleomycin-rat model.	Hesperidin’s effects on pulmonary fibrosis were assessed using micro-computed tomography, histopathology, and a bleomycin-rat model.	Due to its anti-inflammatory, chemical, and antioxidant capabilities, HP therapy causes lung fibrosis while decreasing lipid peroxidation and raising antioxidant status.	[29]
	Bleomycin-induced pulmonary fibrosis in mice.	Hesperidin reduces pulmonary fibrosis by inhibiting lung fibroblast senescence through the IL-6/STAT3 signaling pathway.	Hesperidin decreased senescence-associated-galactosidase-positive cells both in vivo and in vitro, and it can block the IL6/STAT3 signalling pathway.	[33]
Lung cancer	Inhibit cancer cell viability in vitro	Through pinx1, hesperidin prevents lung cancer both in vitro and in vivo.	Hesperidin dramatically enhanced pinx1 protein levels, and blocking pinx1 with a targeted siRNA prevented hesperidin’s protective effects.	[35]
	Rodent model of lung cancer	Citrus juices and pulp with high levels of hesperidin and cryptoxanthin prevent cancer	HPs are a promising cancer chemopreventive agent against the formation of tongue, colon, and lung cancer because they inhibit chemically induced carcinogenesis by detoxifying enzymes, controlling the proliferation and mRNA expression of various cytokines and inflammatory enzymes.	[36]
	Lung initiated with 4-(methylnitrosamino)-1-(3-pyridyl)-1-butanone (NNK) in male A/J mice	Cryptoxanthin and hesperidin-rich mandarin juice’s inhibitory effects on 4-(methylnitrosamino)-1-(3-pyridyl) mouse pulmonary tumorigenesis brought on by 1-butanone	HP decreased the PCNA-positive index in lung cancers while not affecting the PCNA index in lesions with hyperplastic alveolar cells.	[48]
	Swiss albino mice	Hesperidin’s ability to fight cancer and benzo(a)pyrene-induced lung cancer in mice	Lipid peroxides (LPO), carcinoembryonic antigen (CEA), a lung-specific tumour marker, and the serum marker aryl hydrocarbon hydroxylase (AHH) and lactate dehydrogenase were all enhanced by HP. Conversely, these modifications exhibiting a strong anticancer impact in lung cancer were dramatically decreased by hesperidin.	[38]
Acute lung injury	Male BALB/c mice	By preventing the release of HMGB1, hesperidin reduces the acute lung damage brought on by lipopolysaccharide in mice.	HP inhibits the invasion of neutrophils and the synthesis of MCP-1, preventing the transcription and release of HMGB1. Along with neutrophils, macrophages, and myeloperoxidase (MPO) activity, HP cells lowered the elevation of the lung wet-to-dry weight ratio, and therefore lessened lung histological damage	[37]
	In vivo and in vitro model of acute lung inflammation	Effects of hesperidin treatment in a model system of acute respiratory inflammation both in vitro and in vivo.	By regulating the chronic inflammation and redox imbalance, hesperidin may protect the lungs of mice exposed to mechanical ventilation and may even work to stop MV harm.	[41]
	LPS mice in vivo and cell line in vitro	Hesperidin’s in vivo and in vitro innate immunity of endotoxin-induced severe lung damage.	HES inhibits the inflammatory pathways and suppresses the production of ICAM-1, IL-8, IL-1, TNF, IL-6, IL-12, and VCAM-1 in THP-1 and A549 cells.	[40]
	Mouse model of acute lung injury	In a mouse model of acute lung injury, nasal administration of chitosan/ nanoparticles of hesperidin suppresses the cytokine storm syndrome.	In a mouse model of chronic lung illness, nasal administration of HPD/NPS lowers CSS and ALI/ARDS, indicating that anti-inflammatory nanoparticle-based therapeutic approaches may be employed to lessen CSS and ALI in people with inflammatory lung damage.	[49]
Pneumonia	Radiation-induced lung injury male rats	A histopathological study on hesperidin as a radioprotector against radiation-induced lung damage in rats.	HES dramatically reduced lung tissue fibrosis, inflammation, and mast cell proliferation. It also reduced radiation fibrosis and radiation pneumonitis.	[31]
COVID-19	Computational models	Using computational approaches, the medical targets for SARS-CoV-2 are analyzed, and promising medications are found.	One group of andrographolide compounds, hesperidin, showed a strong affinity.	[45]

## Data Availability

No data was used for the research described in the article.
